# Targeting Replication Stress Response Pathways to Enhance Genotoxic Chemo- and Radiotherapy

**DOI:** 10.3390/molecules27154736

**Published:** 2022-07-25

**Authors:** Jac A. Nickoloff

**Affiliations:** Department of Environmental and Radiological Health Sciences, Colorado State University, Fort Collins, CO 80523, USA; j.nickoloff@colostate.edu

**Keywords:** DNA replication stress, DNA damage response, oncogenic stress, genotoxic cancer therapy, targeted therapy

## Abstract

Proliferating cells regularly experience replication stress caused by spontaneous DNA damage that results from endogenous reactive oxygen species (ROS), DNA sequences that can assume secondary and tertiary structures, and collisions between opposing transcription and replication machineries. Cancer cells face additional replication stress, including oncogenic stress that results from the dysregulation of fork progression and origin firing, and from DNA damage induced by radiotherapy and most cancer chemotherapeutic agents. Cells respond to such stress by activating a complex network of sensor, signaling and effector pathways that protect genome integrity. These responses include slowing or stopping active replication forks, protecting stalled replication forks from collapse, preventing late origin replication firing, stimulating DNA repair pathways that promote the repair and restart of stalled or collapsed replication forks, and activating dormant origins to rescue adjacent stressed forks. Currently, most cancer patients are treated with genotoxic chemotherapeutics and/or ionizing radiation, and cancer cells can gain resistance to the resulting replication stress by activating pro-survival replication stress pathways. Thus, there has been substantial effort to develop small molecule inhibitors of key replication stress proteins to enhance tumor cell killing by these agents. Replication stress targets include ATR, the master kinase that regulates both normal replication and replication stress responses; the downstream signaling kinase Chk1; nucleases that process stressed replication forks (MUS81, EEPD1, Metnase); the homologous recombination catalyst RAD51; and other factors including ATM, DNA-PKcs, and PARP1. This review provides an overview of replication stress response pathways and discusses recent pre-clinical studies and clinical trials aimed at improving cancer therapy by targeting replication stress response factors.

## 1. Introduction

Cancer is treated with one or more modalities comprising surgery, genotoxic (DNA damaging) chemo- and radiotherapy, and, increasingly, targeted therapeutics that block oncogenic pathways or enhance immunologic responses in “personalized” therapy. Each treatment modality has advantages and limitations. Surgery can be curative, but it is only effective against local disease, it is invasive, which causes pain and poses risks of infection, and many tumors are unresectable. Genotoxic chemo- and radiotherapy can effectively kill rapidly dividing tumor cells, but some tumor cells are resistant because they divide slowly or are quiescent, such as cancer stem cells and dormant cells [1,2,3]. General genotoxic agents are also limited because they also damage normal tissues, which is dose-limiting. Targeted therapies block oncogenic (cell growth) pathways or enhance immune responses and are therefore cancer-specific, with generally milder side effects than non-targeted therapeutics.

The complexity and built-in redundancy of oncogenic pathways allows resistance to targeted agents to develop by mutation of target genes or by activation of alternative oncogenic cell growth pathways, limiting their long-term effectiveness. For example, the tyrosine kinase inhibitors (TKIs) gefitinib and erlotinib are first-line therapeutics for EGFR-mutant non-small cell lung cancer, but resistance usually develops within two years [4]. This resistance can be overcome by second- and third generation TKIs, but these too soon succumb to resistance [4]. Resistance to agents that target oncogenic/immunologic pathways is driven by genome instability and resultant tumor heterogeneity, two key hallmarks of cancer [5]. In fact, it has been calculated that high mutation rates typically observed in tumors essentially guarantee that several resistance mutations exist before therapy begins [6,7]. In addition to extant resistance mutations, new resistance mutations can arise by several mechanisms, including induction by genotoxic therapeutics, upregulated mutagenesis in response to therapeutic stress (‘adaptive mutability’), and when tumors modulate their microenvironment [8,9,10,11]. Therapeutic resistance is a widespread problem, limiting the long-term efficacy of both targeted and non-targeted therapeutics. Quoting a recent review:

“…regardless of the mechanism of administered therapies (either targeted agents, chemotherapy, or immunotherapy), resistance is a near-universal occurrence.”

[5]

Similar to surgery, radiotherapy is generally limited to treatment of local disease. However, recent advances in our understanding of the abscopal effect, wherein radiation-ablated tumor cells stimulate an immune attack on tumor cells outside the radiation field, has suggested combining radiotherapy with the new generation of immune checkpoint inhibitors to enhance the abscopal effect [12,13,14]. Given a sufficient dose, there is no absolute tumor resistance to radiotherapy [15]. However, as with genotoxic chemotherapeutics, normal tissue tolerance limits radiotherapy doses and many tumors show natural radio-resistance that reflects, for example, slow growing cancer stem cells, dormant cells, hypoxia, enhanced DNA repair, and accelerated tumor cell repopulation [15]. Despite these limitations, genotoxic chemo- and radiotherapy remain critical weapons in the cancer fight.

The effectiveness of genotoxic agents, whether chemical or physical, reflects the sensitivity of tumor cells to DNA damage, and, in particular, DNA double-strand breaks (DSBs), a type of double-strand damage. Along with inter-strand crosslinks induced by bifunctional reactive chemicals such as cyclophosphamide, melphalan, mitomycin C, cisplatin, and psoralen compounds [16], DSBs are among the most cytotoxic DNA lesions, accounting for the widespread use of DSB-inducing agents in cancer therapy [17,18,19,20]. Unlike single-strand lesions, DSBs and inter-strand crosslinks lack an undamaged repair template in the complementary strand in duplex DNA. With double-strand damage, cells are forced to engage a homologous repair template elsewhere in the genome (homologous chromosome, repetitive elements, or sister chromatids in S and G2 phases) to achieve accurate repair, or repair proceeds without a template by error-prone pathways.

Ionizing radiation and most genotoxic chemotherapeutics induce DSBs either directly, or indirectly when single-strand DNA lesions block replicative polymerases, causing replication stress that can lead to replication fork collapse to DSBs [21]. Most DSBs are repaired by canonical non-homologous end-joining (cNHEJ) and homologous recombination (HR). cNHEJ is the dominant DSB repair pathway throughout the cell cycle, while HR is largely restricted to S and G2 phases when sister chromatids are available to serve as homologous repair templates [22]. cNHEJ is fast and efficient, but it is error-prone: repair junctions typically show loss or gain of a few nucleotides [23]. Because HR uses a homologous sequence as a repair template, it is generally accurate. However, any sequence differences (e.g., single-nucleotide polymorphisms) are copied from the donor (undamaged) duplex to the recipient (repaired) duplex, a phenomenon termed gene conversion [24]. In addition, crossovers during HR can cause rearrangements including deletions, inversions, translocations, and large-scale loss of heterozygosity, depending on the linkage and configuration of the interacting homologous sequences [25]. cNHEJ and HR are backed up by alternative NHEJ and single-strand annealing, two intrinsically error-prone pathways that gain importance in cells with defects in cNHEJ and HR factors [26,27,28]. These DSB repair pathways are the effector modules of the complex network of DNA damage sensing, signaling, and repair processes termed the DNA damage response (DDR). The DDR plays a critical role in maintaining genome stability. Key components of the DDR are often dysregulated in tumor cells, as such defects can increase mutation rates and block apoptosis, allowing cells with excessive genomic damage to survive. Ongoing genome instability allows tumor subpopulations to test genetic adaptations that might promote cell growth in the face of intrinsic and extrinsic stressors. Such adaptations can further dysregulate cell growth; promote survival in hostile microenvironments (i.e., low oxygen and nutrient levels, attack by the immune system); gain cell migratory functions that drive invasion and metastasis; develop resistance to intrinsic oncogenic stress reflecting dysregulated DNA replication [29,30]; and develop resistance to extrinsic stress from genotoxic therapeutics [15,31].

Genotoxic chemotherapeutics do not induce DSBs directly, but instead create single-strand DNA lesions (adducts, oxidized bases) that generate DSBs indirectly by blocking DNA replication [20,32]. Although ionizing radiation can induce DSBs directly, most DNA lesions are single-stranded, comprising more than 100 different types including single-strand breaks [33], and these also cause replication stress that results in replication-associated DSBs [34]. Thus, all genotoxic cancer therapeutics induce replication stress, and tumor cells survive these treatments by activating parts of the DDR that manage replication stress. This review focuses on replication stress response factors that have emerged as potential targets to augment the efficacy of genotoxic chemo- and radiotherapy.

## 2. Key Features of the Cellular DNA Damage Response

Cellular responses to DNA damage can be described in three phases: (i) lesion recognition (sensing), (ii) signaling through phosphorylation cascades and other signal amplification systems, and (iii) activation of checkpoint responses that arrest the cell cycle; promote DNA repair, promote repair and restart stalled or collapsed replication forks; and trigger programmed cell death by apoptosis, autophagy, or other death pathways when damage is excessive [35,36,37]. The DDR is regulated by a trio of kinases in the phosphatidyl inositol 3’ kinase-related kinase (PIKK) family, DNA-PKcs (DNA dependent protein kinase catalytic subunit), ATM (ataxia telangiectasia mutated), and ATR (ATM and RAD3-related). These are very large proteins (300 to 470 kDa) with common structural elements, including FRAP-ATM-TRRAP (FAT) and FAT C-terminal (FATC) domains that flank and regulate kinase domains, and HEAT repeats that mediate protein–protein interactions [35]. These PIKKs are quite promiscuous: ATM and ATR phosphorylate at least 900 targets in >700 proteins, typically at consensus SQ or TQ motifs [38], although non-consensus targets have been identified [39]. In response to DSBs and replication stress, activated PIKKs are themselves targets, via auto- and trans-phosphorylation.

**Figure 1:**
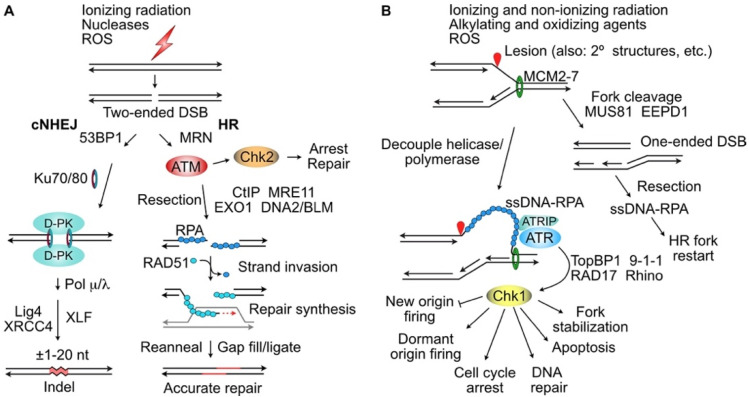
DNA-PK, ATM and ATR roles DSB repair by cNHEJ, HR, and replication fork restart. (**A**) Two-ended (frank) DSBs are shunted to cNHEJ if resection is blocked by 53BP1 (and RIF1), or to HR when resection is promoted by MRN and signaling through ATM. Ku70/80 binds unresected ends and recruits DNA-PKcs (D-PK) which tethers ends, and promotes end alignment, template-dependent and -independent nucleotide addition by Pol λ and Pol μ, respectively, and ligation by Lig4-XRCC4 and XLF to yield indel repair products. ATM phosphorylates/activates Chk2 which promotes cell cycle arrest and repair. ATM stimulates DSB repair by HR by phosphorylating targets that promote limited end resection by CtIP and MRE11, and extensive resection by EXO1 and DNA2-BLM. The resulting 3′ ssDNA extensions are bound by RPA, which is replaced by the HR recombinase RAD51 that catalyzes invasion of homologous duplexes (typically sister chromatids). Eviction of RAD51 allows repair synthesis to extend the invading strand across the DSB. The extended end releases from the donor duplex (grey) and anneals to complementary ssDNA on the opposite side of the DSB. Gap filling and ligation complete accurate HR repair. (**B**) Replication forks blocked by single-strand lesions or secondary structures, or stalled by nucleotide depletion or DNA polymerase inhibitors, causes decoupling of polymerase from the MCM helicase, which spools out ssDNA that is bound by RPA. ATRIP mediates the interaction between ssDNA-RPA and ATR, which leads to Chk1 activation that phosphorylates targets that effect several DNA damage checkpoint responses. Blocked or stalled forks may be cleaved by nucleases MUS81 or EEPD1 to yield one-ended DSBs that are resected to promote HR-mediated fork restart and prevent genome rearrangements due to cNHEJ rejoining of one-ended DSBs to ends of other DSBs.

There is significant crosstalk among the PIKKs, as certain autophosphorylation targets may be phosphorylated by other PIKKs under specific conditions [40,41,42]. Replication protein A (RPA) has several residues targeted by multiple PIKKs, demonstrating further crosstalk [43,44]. Despite this crosstalk, the primary roles of DNA-PKcs and ATM are to promote the repair of two-ended (frank) DSBs by cNHEJ and HR, respectively (Figure 1A). The primary roles of ATR are to regulate replication, respond to replication stress by protecting stalled replication forks by preventing replisome dissociation and fork collapse to DSBs [45,46], and to promote HR-mediated repair and restart of collapsed forks [47,48].

PIKK kinases are activated in response to DNA damage by complex mechanisms. Phosphorylation of ATM was suggested to contribute to activation of its kinase through a feed-forward mechanism [49,50,51], although later studies in cells and mice questioned this conclusion [52,53,54,55]. Phosphorylation of DNA-PKcs is an important step in DSB repair by cNHEJ [39,56,57]. ATR is activated/phosphorylated in response to single-stranded DNA (ssDNA) coated with the trimeric RPA complex [58,59], a process mediated by the ATR interacting protein ATRIP [60]. ssDNA forms naturally and is bound by RPA at replication forks when the MCM helicase unwinds DNA ahead of the fork. Constitutive, low-level ATR activation by physiologic levels of ssDNA-RPA regulates replication origin firing to achieve a relatively stable number of active replication forks during the S phase [61]. When a replisome encounters a blocking lesion, the MCM helicase can decouple from the leading-strand polymerase, producing long segments of ssDNA-RPA, and this causes full activation of ATR (Figure 1B). Recent evidence indicates that ATR and ATRIP form a tetrameric complex with two ATR and two ATRIP subunits, and that ATR activation depends on either TopBP1 (in response to DNA damage) or ETAA1 (regulating ATR during normal DNA replication and mitosis) [62,63,64].

## 3. Sources of DNA Replication Stress

Cells experience replication stress continuously from spontaneous DNA damage, difficult-to-replicate DNA sequences, and collisions between replication and transcription machineries. Nearly all DNA lesions block replicative polymerases, causing fork stalling, including single-strand breaks, DSBs, pyrimidine dimers, adducted and oxidized bases, and broken rings. DNA lesions cause localized replication stress, but genome-wide stress can be induced by the depletion of nucleotide pools with hydroxyurea, and by aphidicolin, a DNA polymerase inhibitor [32,65,66]. Spontaneous DNA damage results from normal DNA lability, ribonucleotide misincorporation, and reactive oxygen species (ROS), including hydroxyl radicals (OH⦁) and superoxide anions (O_2_**⦁**^−^) generated during mitochondrial oxidative metabolism [67,68]. ROS causes oxidative DNA damage, abasic sites, single-strand breaks, and other lesions [69] that have been linked to mutagenesis, cellular senescence, redox imbalance, and aging [70]. For the most part, highly transcribed genes are arranged within chromosomal replication domains to minimize collisions between replication and transcription, but chromosomal rearrangements, including translocations and inversions, can upset this order and increase replication stress. Highly transcribed ribosomal RNA genes, telomeres, and fragile sites are also sources of replication stress [71,72,73,74,75]. Certain DNA sequences are difficult to replicate, and these are additional endogenous sources of replication stress, including G-rich sequences in which stable R-loops [76,77,78], and G-quadruplex structures can form [71,79,80,81,82]. Replication stress is also caused by induced DNA lesions from incidental exposures to exogenous genotoxic chemicals in air, water, and food; from ultraviolet light (primarily from sun exposure) which causes damage directly and through ROS generation; from intentional exposures to genotoxic chemotherapeutics and ionizing radiation during cancer therapy; and by topoisomerase I inhibitors that induce replication stress by causing DNA overwinding ahead of the replication fork, driven by the MCM helicase [83,84,85].

## 4. Repair and Restart of Stressed Replication Forks

Several DNA damage tolerance pathways exist that allow lesions to be bypassed and replication to continue, leaving the lesions to be repaired at a later time. One tolerance mechanism is direct lesion bypass by less structurally constrained translesion DNA synthesis (TLS) polymerases. These include members of the DNA polymerase Y-family, Rev1, Pol η, Pol ι and Pol κ; Pol θ in the A-family, Pol ζ in the B-family, and PRIMPOL [86,87]. Reflecting their flexibility in catalyzing synthesis on damaged DNA templates, TLS polymerases are error-prone, and therefore represent important drivers of mutagenesis and carcinogenesis [88,89,90,91]. Pol β with a primary role in base excision repair, is another error-prone polymerase that has been linked to cancer predisposition and is thus accurately described as a tumor suppressor [92,93,94]. Other lesion tolerance pathways are repriming by PRIMPOL (which results in under-replicated single-strand gaps [95]), HR-mediated template switching, and passive rescue of a stalled fork by an adjacent fork [21,96].

**Figure 2:**
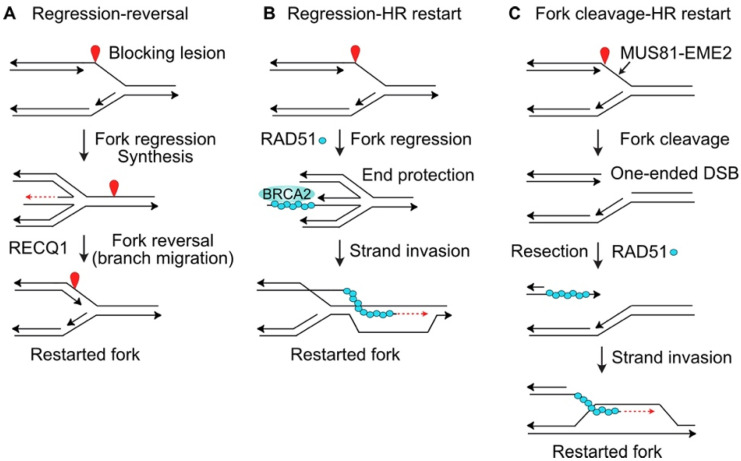
Fork restart mechanisms. (**A**) Blocked forks may regress to form 4-strand, Holliday junction-like structure and a one-ended DSB. DNA synthesis (dashed/red arrow) using newly replicated strand as template allows the blocked end to extend beyond the blocking lesion. The fork is restarted once RECQ1 drives fork reversal by branch migration. (**B**) The one-ended DSB at regressed forks may be protected by RAD51, BRCA2, and other factors. Fork restart is mediated by RAD51-ssDNA filament invasion of the unreplicated duplex DNA. (**C**) Stalled forks may be cleaved by MUS81-EME2 (or EEPD1, not shown), creating a one-ended DSB. Resection creates ssDNA that is first bound by RPA and then RAD51, and the RAD51-ssDNA filament catalyzes strand invasion to restart the fork, similar to panel (**B**).

Stalled replication forks may also be restarted by at least three mechanisms that involve fork regression or fork cleavage. A blocked fork may regress, creating a 4-strand branched structure that resembles a Holliday junction, and this allows the blocked strand to anneal to the nascent strand of the sister chromatid, and prime synthesis using the nascent strand as a template (Figure 2A). If this synthesis extends far enough, the regressed fork can reverse in a reaction catalyzed by RECQ1 [83], reestablishing a functional replication fork, and bypassing the blocking lesion (Figure 2A). Regression of a stalled fork also produces a structure with a one-ended DSB that is prone to attack by nucleases. Cells protect these ends with two key HR proteins, RAD51 and BRCA2 (Figure 2B), along with a host of other factors including RIF1, FANCA, FANCC, FANCD2, FANCG, ABRO1, VHL, RADX, and BOD1L [97,98,99]. There are at least two distinct fork regression mechanisms, one promoted by HLTF, SMARCAL1 and ZRANB3, and another by FBH1; 53BP1 is a fork protection factor for FBH1-remodeled forks [97]. The RAD51 filament in the protected regressed fork can catalyze strand invasion to reestablish a functional replication fork (Figure 2B). A third class of fork restart pathway involves cleavage of the stalled fork by branched structure-selective nucleases MUS81 (with its EME2 cofactor) or EEPD1 [100,101,102,103,104] (Figure 2C). Cleavage of a stalled fork creates a one-ended DSB that poses a significant risk of large-scale genome rearrangement if cNHEJ mediates rejoining with another DSB elsewhere in the genome (i.e., at another broken fork or one of the ends at a frank, two-ended DSB). To avoid genome destabilization by cNHEJ-mediated mis-repair of the one-ended DSBs at cleaved forks, the EEPD1 pathway includes an important safeguard. EEPD1 interacts with and promotes EXO1-mediated end-resection of the one-ended DSB to inhibit cNHEJ and promote accurate fork repair and restart by HR [21,102]. An analogous system to promote the resection of one-ended DSBs induced by MUS81-EME2 has yet to be described. MUS81 is a 3′ nuclease first identified in Saccharomyces cerevisiae [105] and this forces invasion of the one-ended DSB into the discontinuous lagging strand duplex. In contrast, the 5′ nuclease EEPD1 evolved more recently in chordates/vertebrates, and this mode of cleavage allows invasion by the resected end into the continuous, leading strand duplex. The EEPD1 cleavage mechanism may speed fork restart and thereby reduce the opportunity for stressed forks to assume toxic structures, promoting both genome stability and cell survival under stress [43].

## 5. Targeting Replication Stress Signaling and Fork Repair/Restart Pathways in Cancer Therapy

The importance of replication stress responses in cells exposed to genotoxic chemo- or radiotherapy has prompted considerable research focused on how tumor cells might be selectively killed by combined treatments with genotoxins and agents targeting DDR and replication stress factors. TLS polymerases and other DNA damage tolerance pathways contribute to cancer chemoresistance, hence key factors in these pathways have been explored as potential targets to augment genotoxic therapies. These targets are not discussed further here as several excellent treatises have been published on this topic [87,106,107,108,109,110,111,112,113,114,115]. Table 1 summarizes the replication stress factors targeted in cancer therapy, discussed below.

### 5.1. Targeting Downstream DNA Damage Checkpoint Factors (Chk1, Chk2, Wee1)

Many DDR targets along the DNA damage sensing-signaling-repair continuum have been explored as cancer therapeutic targets. In the 1990s and early 2000s, there was considerable excitement about combining genotoxic chemo- or radiotherapy with inhibitors of checkpoint effector kinases. The general idea was that by inhibiting effector checkpoint kinases Chk1, Chk2, or Wee1, cells experiencing genotoxic stress would fail to arrest, and continued cell cycle progression in the face of significant DNA damage would lead to cell death by mitotic catastrophe or other mechanisms. This prompted the early development of Chk1 inhibitors (Chk1i) including UCN-01. Despite success in preclinical studies, in a Phase I trial, UCN-01 had a long half-life but limited bioavailability due to high avidity to plasma α1-acid glycoprotein, and serious side effects were observed when doses were increased to exceed the plasma binding capacity [116]. By 2013, at least 12 additional Chk1i were developed, but like UCN-01, many were cross-inhibitory with other targets (e.g., Chk2, CDK1, VEGFR2, PIM1) and only one, LY2603618, combined with the antifolate antineoplastic drug Pemetredex, reached a Phase I/II trial [117]. The severe side effects of UCN-01 may reflect the rather broad impact that Chk1 has on cellular functions, including replication initiation, replication fork stabilization, cell cycle progression, DNA repair, and apoptosis (Figure 1B). Alternatively, the disappointing results with early Chk1i may reflect their lack of specificity. More potent/specific Chk1i have been developed and several have been tested in recent Phase I or Phase II trials in mono- or combination therapies. The Chk1i GDC-0575 (±gemcitabine) had manageable side effects that were primarily hematologic, but GDC-0575 showed limited antitumor activity against advanced solid tumors [118]. A phase I trial with the Chk1i MK-8776 (SCH 900776), also in mono- or combination therapy with gemcitabine against advanced solid tumors, had adverse effects on cardiac function and caused abdominal pain, despite minimal clinical benefit (NCT00779584). In a Phase II trial, MK-8776 tested as an adjunct to AraC (Cytarabine) treatment for relapsed acute myeloid leukemia also showed adverse effects on cardiac function and other organ toxicities but no clinical benefit beyond that of AraC alone (NCT01870596). LY2606368 (Prexasertib) is a Chk1i that causes mitotic catastrophe (also termed replication catastrophe) in vitro and showed antitumor activity in preclinical animal studies [164], yet in several Phase I/II trials, LY2606368 has shown limited clinical benefit with occasional serious side effects [120,121,122,123]. Wee1 is a checkpoint kinase in the ATR/Chk1/Wee1 pathway that negatively regulates the G2/M transition, among other functions [165]. The Wee1i MK1775 (AZD1775, Adavosertib) showed a significant increase in progression free survival in a Phase II trial of ovarian cancer treated with Paclitaxel and carboplatin ± MK1775 [124].

### 5.2. Targeting the Upstream DNA Damage Checkpoint Kinase ATR and Its Activation Partner TopBP1

Given the propensity of serious side effects and limited clinical benefits to date for Chk1i, it may seem counterintuitive that targeting ATR, which acts upstream of Chk1, would have fewer adverse effects on normal tissues. Nonetheless, potent and relatively specific ATRi have been developed and several have been tested in mono- or combination therapy in Phase I/II trials. The ATRi BAY1895344 has shown initially promising results in a Phase I trial against advanced solid tumors, with ~20% of patients showing partial responses and nearly 40% showing stable disease. Although adverse effects with BAY1895344 were common, including hematologic problems, fatigue, and nausea, these were manageable [125]. The ATRi AZD6738 (Ceralasertib) was well tolerated in combination with carboplatin in a Phase I trial that also showed moderate clinical benefit [126]. Interestingly, in both of these ATRi trials, patients who responded had DDR defects, including loss of ATM. These results should motivate further studies with ATRi and other DNA damage checkpoint inhibitors in patients with DDR defects to further develop personalized therapies.

TopBP1 has many binding partners and functions in a variety of cellular processes [166], including itself, topoisomerase IIβ, p53, and RAD51 [167], and it plays a critical role in ATR signaling to Chk1 (Figure 1A) [168]. TopBP1 expression is frequently increased in osteosarcoma and other sarcomas, and this correlates with poor prognosis [169]. Interestingly, low or moderate levels of TopBP1 correlate with normal ATR/Chk1 activation during stress, but TopBP1 overexpression has an inhibitory effect on ATR/Chk1 activation [170], which, in theory, would promote the resistance of tumor cells to DNA damage by suppressing apoptosis. Because TopBP1 functions depend on protein–protein interactions, specific inhibitors that block these interactions are of interest. The cell viability dye calcein acetoxymethyl ester (calcein AM) was shown to interfere with TopBP1 oligomerization and p53 binding, resulting in the reactivation of apoptosis and interference with mutant/oncogenic p53 [127]. Calcein AM showed antitumor activity against breast cancer xenografts [127] and it increased the antitumor activity of the topoisomerase inhibitor doxorubicin against lung tumor xenografts [128]. To date no TopBP1 inhibitors have advanced to clinical trials.

### 5.3. Targeting Replication Stress Nucleases (CtIP, MUS81, EEPD1, Metnase)

Several nucleases play key roles in the replication stress response [43]. CtIP is a nuclease and key factor in the early steps of DNA end resection at DSBs, functioning with BRCA1 in both end resection and the protection of stressed replication forks [171,172,173]. It thus plays an important role in creating the ssDNA-RPA substrate required for ATR activation (Figure 1B). A recent study demonstrated that a peptide mimic that blocks CtIP tetramerization is a specific CtIPi that impairs DSB repair, interferes with stressed fork protection, sensitizes cells to DNA damage and PARP1i, and is cytotoxic to BRCA1-defective cells [129]. CtIP is also a target of a lncRNA (lnc15.2)-encoded micropeptide termed PACMP. PACMP stabilizes CtIP by blocking its ubiquitination, and it also promotes poly(ADP)ribosylation of substrates by PARP1 [130]. siRNA knockdown of lnc15.2 causes hypersensitivity to PARP1i, ATRi, CDK4/6i, the TopoI inhibitor camptothecin, the TopoII inhibitor epirubicin, and radiation, and it has antitumor activity against breast tumor xenografts [130]. Together, these results warrant studies to further test the antitumor effects of CtIPi in preclinical and clinical trials.

Several nucleases that process stressed replication forks are being explored as therapeutic targets. MUS81 cleaves stalled replication forks to initiate HR-mediated fork restart [100], and it has shown promise as a target in preclinical studies [131,174]. EEPD1 also cleaves stalled replication forks [103,104] and given that MUS81 and EEPD1 cleave forks with different polarity, combining MUS81i and EEPD1i may be potent approach to augment the cytotoxic effects of replication stress. Metnase is a nuclease that promotes replication fork restart, but it does not cleave stalled forks [103]. Metnase nuclease can be inhibited by the frequently administered antibiotic Ciprofloxacin [132], suggesting a safe and potentially effective means to augment genotoxic cancer therapy, perhaps in combination with drugs targeting other DDR factors.

### 5.4. Targeting DSB Repair Proteins (RAD51, PARP1)

RAD51 plays a central role in HR, and HR plays a critical role in fork protection and the accurate repair and restart of collapsed replication forks (Figure 2B,C). RAD51 is frequently overexpressed in cancer cells [175], thus RAD51 has emerged as a potentially useful target in cancer therapy. RI-1 is a RAD51i that inhibits RAD51-RAD51 interactions that are important for RAD51-ssDNA nucleoprotein filament formation, the key complex that catalyzes HR [176]. RI-1 has been shown by two groups to radiosensitize glioblastoma cells [133,134] and it enhances the chemosensitivity of glioblastoma cells to genotoxic damage by alkylating agents [135]. The RAD51i B02 interferes with RAD51-mediated strand invasion [177], and B02 sensitizes glioblastoma cells to alkylating agents [135], and it sensitizes multiple myeloma cells to doxorubicin [136]. Tumors with HR defects, such as BRCA1- and BRCA2-defective breast cancer are sensitive to PARP1i because PARP1 promotes the repair of single-strand lesions that block replication forks and therefore require HR to repair/restart these forks [178,179]. The original B02 compound has been modified into a B02-isomer with greater potency, and the HR defect induced by B02-isomer shows strong synergistic sensitization of tumor cells with PARP1i [180].

PARP1 adds poly(ADP)ribose (PAR) groups to numerous target proteins. PARylation of repair factors is seen in response to DNA damage processed by base excision repair, nucleotide excision repair, single-strand break repair, and DSB repair by cNHEJ, alternative NHEJ, and HR [181]. PARP1i induces replication stress by at least two mechanisms: PARP1i increases the load of unrepaired endogenous DNA lesions (analogous to increased lesion load by exogenous genotoxins), and it blocks replication directly by trapping PARP1 on DNA [182]. In addition, recent studies have revealed new roles for PARP1 in chromatin remodeling. DNA repair occurs within the chromatin environment, so it is no surprise that proteins that modify or remodel chromatin influence DNA repair [183,184,185,186,187,188,189,190] and replication stress responses [191,192]. PARP1 has emerged has an important factor in chromatin modeling, and PARylation of both repair factors and chromatin promote the recruitment of repair factors to damaged sites [181,193].

Normal cells cope with PARP1i-induced replication stress by marshaling HR to repair and restart collapsed replication forks, but in cancer cells with HR defects, PARP1i are synthetically lethal [194,195]. First exploited in cancer cells with mutant BRCA1 or BRCA2, PARP1i are potentially valuable against tumors with defects in other HR proteins due to inherited germline mutations or sporadic somatic mutations. The PARP1i olaparib was the first targeted therapeutic approved to treat BRCA-defective ovarian cancer, and PARP1i are being explored to treat BRCA-mutant breast, ovarian, prostate, and pancreatic cancers [196,197,198,199]. In 2020 the FDA approved two PARP1i, olaparib and rucaparib to treat metastatic castration-resistant prostate cancer, and it approved olaparib to treat eligible patients with pancreatic cancer. Because PARP1 plays many roles in DNA repair, replication stress responses, and chromatin remodeling, additional uses for PARP1i in cancer monotherapy, combination therapy, and maintenance therapy continue to be explored (Table 1) [157,158,159,160,161,162,163].

### 5.5. Exploiting Synthetic Lethality of TKIs Targeting Activated Oncogenes and ATM Inhibitors

When cancer cells activate oncogenes that promote growth, an important consequence is oncogenic stress, also known as oncogenic replicative stress [29]. Oncogenic stress reflects dysregulated replication initiation and progression, including mis-timed origin firing. Many drugs targeting activated oncogenes have been developed and brought to clinical practice, including drugs that target activated HER2/ERBB2, ALK, KRAS, BRAF, or EGFR [200,201,202,203,204,205]. It was recently shown that blocking oncogenic pathways with targeted therapeutics (TKIs) causes moderate stress which results in sublethal DSBs induced by caspase-activated DNase (CAD; also known as DFF40 and DFFB) [156]. When fully activated during apoptosis, CAD is responsible for digesting the genome into ~180 bp “ladders” by cleaving linker DNA between nucleosomes [206]. Although cells survive these TKI/CAD-induced DSBs, their repair depends on ATM, thus ATMi kills cells treated with various TKIs targeting activated oncogenes [156]. This effect appears to be general, as ATMi kills tumor cells treated with cognate TKIs targeting different activated oncogenes (EGFR, ALK, KRAS, and BRAF) in different tumor types (lung, pancreatic, melanoma, and acute myeloid leukemia). Interestingly, TKI + ATMi is cytotoxic even in cells that have gained resistance to the cognate TKI, suggesting ATMi may prove beneficial to patients with TKI-resistant tumors [156]. This TKI + ATMi effect represents a novel approach to exploit replication stress (oncogenic stress) and DDR inhibition to selectively target cancer cells.

## 6. Perspectives

The DDR has emerged as a rich source of targets that may be exploited to augment the cytotoxic effects of genotoxic chemo- and radiotherapy that cause replication stress. The value of DDR targeting became abundantly clear with the discovery in 2005 that PARP1i selectively kills tumor cells with HR defects [194,195], and this has spurred research seeking additional tumor-specific vulnerabilities related to the DDR, including those based on the addiction of tumor cells to DNA repair [207]. Because DDR functions are important in normal cells, cross-toxicity presents a barrier to effective eradication of tumor cells. The unacceptable side effects caused by early Chk1 inhibitors highlight these challenges. Nonetheless, moving the target up- or downstream along DDR signaling and repair pathways has shown success in preclinical studies, and, as discussed above, several DDR inhibitors are in clinical trials. Each DDR factor presents unique targeting opportunities, from kinase inhibitors and nuclease inhibitors to inhibitors of protein–protein interactions, as evidenced by the RAD51i RI-1. Interactions between PIKKs and other proteins are mediated by their HEAT repeats, so blocking these interactions may prove effective in inhibiting the DDR. It took nearly a decade from the discovery of PARP1i effects on BRCA-defective cancer cells to FDA approval of olaparib in combination therapy of suspected BRCA-deficient/defective ovarian cancer. It took 15 years for olaparib to be approved by the FDA as an ovarian cancer monotherapy. Efforts focused on inhibitors of PARP1, and other replication stress factors, are growing research fields. Given the central role of replication stress in genotoxic cancer therapy, the complexity of the DDR, and the challenges presented by tumor heterogeneity and therapeutic resistance, opportunities to advance replication stress targeting in basic, preclinical, and clinical arenas will continue far into the future.

## Figures and Tables

**Table 1:** Replication stress targets in preclinical development and clinical trials.

Target Protein	Inhibitor	Recent/Ongoing Clinical Trials *	Tumor/Cell Targets **	References
Chk1/Chk2	UCN-01		Solid tumors, leukemia	[116]
	LY2603618	NCT01341457	Solid tumors	[117]
	GDC-0575	NCT01564251	Solid tumors, lymphoma	[118]
	MK-8776	NCT01870596	Acute myeloid leukemia	[119]
		NCT00779584	Solid tumors, lymphoma	
	LY2606368	NCT02735980	Lung cancer	[120]
		NCT02778126	Solid/CNS tumors	[121]
		NCT02203513	TNBC	[122]
		NCT01115790	Advanced solid tumors	[123]
Wee1	MK1775	NCT01357161	Ovarian cancer	[124]
ATR	BAY1895344	NCT03188965	Advanced solid tumors	[125]
	AZD6738	NCT02264678	Advanced solid tumors	[126]
TopBP1	Calcein AM		Breast, lung xenografts	[127,128]
CtIP	SP(18–28)		Breast cancer cells	[129]
PACMP	siRNA-lnc15.2		Breast, ovarian, lung, OS, stomach cancer cells	[130]
MUS81	siRNA-MUS81		Gastric cancer cells	[131]
EEPD1	siRNA-MUS81		Lung, OS, fibrosarcoma, cervical cancer cells	[103,104]
Metnase	Ciprofloxacin		Embryonic kidney cells	[132]
RAD51	RI-1		Glioblastoma, glioma cells	[133,134,135]
	B02		Glioblastoma, multiple myeloma cells	[135,136]
PARP1	Olaparib	NCT03531840	Mesothelioma	[137]
		NCT03402841	Ovarian cancer	[138]
		NCT03286842	Breast cancer	[139]
		NCT02983799	Ovarian cancer	[140]
		NCT02789332	Breast cancer	[141]
		NCT02734004	Breast cancer	[142]
		NCT02477644	Ovarian cancer	[143]
		NCT01513174	Lung cancer	[144]
		NCT02282020	Ovarian cancer	[145]
	Rucaparib	NCT02952534	Prostate cancer	[146]
		NCT02042378	Pancreatic cancer	[147]
		NCT01891344	Ovarian cancer	[148]
		NCT01074970	Ovarian, solid tumors	[149]
		NCT01891344	Ovarian cancer	[150]
	Niraparib	NCT03759600	Ovarian cancer	[151]
		NCT04475939	Lung cancer	[152]
		NCT02657889	TNBC	[153]
		NCT02354131	Ovarian cancer	[154]
		NCT02354586	Ovarian cancer	[155]
ATM	AZD0156	NCT02588105	Advanced solid tumors	[156]
ATM/ATR	AZD1390	NCT03215381	Healthy volunteers	[156]

* Clinical trials ongoing (actively enrolling) or completed since 2015. Most trials include combination therapies with the indicated inhibitor. For PARP1i, only completed Phase II/III trials with peer-reviewed publications since 2015 are listed: data from ClinicalTrials.gov. Several recent reviews provide additional information about the numerous PARP1i clinical trials [157,158,159,160,161,162,163]. ** CNS, central nervous system; TNBC, triple negative breast cancer; OS, osteosarcoma.

## Abbreviations

Calcein AM, calcein acetoxymethyl ester; CAD, caspase-activated deoxyribonuclease; cNHEJ, canonical non-homologous end-joining; DDR, DNA damage response; DSB, DNA double-strand break; FAT, FRAP-ATM-TRRAP domain; FATC, FAT C-terminal domain; HEAT, protein–protein interaction domain named for Huntingtin, EF3, PP2A, and TOR1; HR, homologous recombination; PAR, poly(ADP)ribose; PIKK, phosphatidyl inositol 3’ kinase-related kinase; ROS, reactive oxygen species; ssDNA, single-stranded DNA; TKI, tyrosine kinase inhibitor; TLS, translesion DNA synthesis.
